# Associations of Dietary Patterns and Incident Type 2 Diabetes in a Community Population Cohort From Southwest China

**DOI:** 10.3389/fpubh.2022.773172

**Published:** 2022-02-03

**Authors:** Yanhuan Wang, Lina Xu, Na Wang, Ling Zhu, Fouxi Zhao, Kelin Xu, Tao Liu, Chaowei Fu

**Affiliations:** ^1^National Health Commission Key Laboratory of Health Technology Assessment, School of Public Health, Fudan University, Shanghai, China; ^2^Guizhou Center for Disease Control and Prevention, Guiyang, China

**Keywords:** dietary patterns, factor analysis, incidence, type 2 diabetes, China

## Abstract

**Background:**

The prevalence of type 2 diabetes (T2D) is rising rapidly worldwide, but there are scant empirical data on the association between diet and diabetes in Southwest China.

**Methods:**

In this prospective community-population cohort study from Guizhou Province, China since 2010, 7,023 eligible adults were included. Dietary information was obtained by face-to-face interviews with a semi-quantitative food frequency questionnaire, and dietary patterns were derived by factor analysis. The hazard ratios (HR) and 95% confidence intervals (95% CI) were estimated for the associations between various dietary patterns and incident T2D risk by cox proportional hazard model.

**Results:**

Until 2020, a total of 749 new T2D cases were identified during the average follow-up of 7.05 years and the incidence was 14.75/1,000 person-years. Two main dietary patterns from the food frequency questionnaire were identified by factor analysis, i.e., vegetable-grain pattern and junk food pattern. In the multivariate analysis, 28 and 20% lower risks of T2D were observed at the low intake of junk food pattern (HR: 0.72, 95% CI: 0.61, 0.87) and the high intake of vegetable-grain pattern (HR: 0.80, 95% CI: 0.67, 0.95) after adjustment for potential confounding factors, compared with the medium intake of such patterns, respectively. Positive linear relationships were found between fasting plasma glucose (FPG) at follow-up and its change with junk food pattern, while there were inverse linear associations with vegetable-grain pattern.

**Conclusion:**

Higher adherence to vegetable-grain patterns and lower adherence to junk food patterns significantly lowered T2D incidence among the population in Southwest China. Moving toward a healthier dietary model deserves more attention to develop interventions for the prevention of T2D.

## Introduction

The incidence and prevalence of diabetes continue to rise unprecedentedly, and this epidemic has accelerated in low-income and middle-income countries (LMICs) (1). China has the highest number of people with diabetes in the world (114.4 million in 2020), with type 2 diabetes (T2D) accounting for more than 90% of patients with diabetes and leading to a huge burden on healthcare systems (2). Increased understanding of risk factors for T2D can help reduce morbidity and mortality.

Transition in food consumption among Chinese residents has played an important role in the fast increase of T2D (3) and diet has been identified as a modifiable factor for occurrence and progression in diabetes by many researchers (4, 5). The Guideline for Prevention and Treatment of T2D in China (2020 Edition) also pointed out the necessity to set reasonable treatment targets for medical nutrition (6). Nowadays, dietary pattern analysis has emerged as an alternative and complementary approach to examine the relationship between diet and diseases. It considers the interaction and joint effects of different food items and examines the effects of the overall diet. Several studies have suggested that dietary patterns derived from factor analysis were valued in the nutritional epidemiology of diabetes but different patterns with positive or negative health effects such as the anti-inflammatory dietary pattern were applied (4, 7–9). Such disparities in dietary patterns may be partly due to the diversity in available food, food cooking, and eating habits over study populations in the context of various times, race, and culture, which meant that it should benefit the prevention and control of diabetes to understand particular recommended diet patterns for the local people.

The “refined grains and meat” dietary pattern and the “oil and salt” dietary pattern were reported to increase T2D risk in the Xinjiang Region (10), and individuals with the Western dietary pattern had an elevated risk of T2D in Zhejiang Province, China (11). Even in mainland China, dietary patterns varied over provinces from Northeast China to Southwest China. So far, there were no empirical data on the association between dietary patterns and diabetes in Southwest China. Several specialized dietary patterns were characterized by using factor analysis and their associations with T2D incidence were explored in this community population cohort from Southwest China.

## Methods and Materials

### Study Population

The Guizhou Population Health Cohort Study (GPHCS) as a prospective community-based population cohort started from 2010 to 2012 with 9,280 adults in Guizhou province, China. Subjects were recruited from 48 townships of 12 counties (or districts) based on a multistage proportional stratified cluster sampling method. The inclusion criteria were as follows: (1) aged 18 years or above; (2) living in the study region and having no plan to move out; (3) completing survey questionnaire and blood sampling; (4) signing the written informed consent. Baseline information was collected using a structured questionnaire on demographic characteristics, lifestyle, and health conditions while dietary data were measured by the semi-quantitative food frequency questionnaire through face-to-face investigation. The questionnaires were designed by the Chinese Center for Disease Control and Prevention (12) and applied in China's chronic disease surveillance (2010). Venous blood samples were obtained to measure fasting plasma glucose (FPG) for at least 8 h fasting. A 2-h oral glucose tolerance test (OGTT) with 75 g of glucose was carried out for participants. Anthropometric measurements including height and body weight, FPG, OGTT, and HbA1c were measured by trained investigators and doctors.

This cohort was followed up by a repeated investigation from 2016 to 2020 with a follow-up rate of 88% (*n* = 1,117 loss to follow-up). All deaths were identified through the Death Registration Information System and Basic Public Health Service System. We further excluded participants who left >1 food item blank (*n* = 277), had a confirmed diagnosis of diabetes (*n* = 665) reported at baseline, or missed the date of diagnosis of diabetes (*n* = 18) at follow-up. In total, 7,023 participants were eligible for the analysis (Supplementary Figure 1). This study was approved by the Institutional Review Board of Guizhou Province Center for Disease Control and Prevention (No. S2017-02). All subjects provided written informed consent at enrollment.

### Assessment of Dietary, Type 2 Diabetes, and Covariates

For dietary, subjects were asked about the frequency and consumption of 16 aggregated food groups in one recent year. Food intake frequency includes “times per day,” “times per week,” “times per month,” and “times per year” with the average intake per time. Except for egg that was expressed in “number per day” and carbonated soft drinks and other soft drinks in “ml per day,” the unit of other food groups was “50 g per day.” Before data analysis, the average daily intake of each food group was estimated by multiplying the intake frequency and single intake converted into continuous variables and dividing by 1, 7, 30, or 365, respectively. T2D was defined if subjects met either of the following criteria: (1) self-reported doctor diagnosis of diabetes or use of anti-diabetic medications confirmed by medical record review; (2) FPG ≥7.0 mmol/L; (3) OGTT ≥11.1 mmol/L; (4) HbA1c ≥6.5% (13). Impaired fasting glucose (IFG) was defined biologically as a fasting plasma glucose concentration from 6.1 to 7.0 mmol/L. The body mass index (BMI) was calculated by dividing the weight in kilograms by the square of the height in meters and categorized as lean (<18.5), normal (18.5–23.9), overweight (24–27.9), obesity (≥28 kg/m^2^) with the Guidelines for Prevention and Control of Overweight and Obesity in Chinese Adults (14).

### Statistics Analysis

Factor analysis with the orthogonal rotation procedure varimax was applied to analyze the intake of 16 food groups and generate main dietary patterns for this study population. The dietary pattern was interpreted and named according to the factor loading matrix, and the scores of dietary patterns were calculated and categorized into tertiles of low, medium, and high. Finally, four factors were obtained with eigenvalues>1 and two factors of them were occupied for this analysis (15, 16). The intake of each food group was described as tertiles of dietary patterns (Supplementary Table 1). The first factor was characterized by the high factor load (0.34–0.54) of fried food, soft drinks, and desserts and named as junk food pattern. The second factor was characterized by the high factor load of vegetable and grain (0.54 and 0.42) and named vegetable-grain pattern. In the medium category of vegetable-grain pattern, the per capita daily vegetable (401.19 g) consumed by participants met the recommended quantity (300–500 g) by the Dietary Guidelines for Chinese Residents (2016 Edition) (17), and therefore the medium intake is used as the reference group.

The Chi-square test for categorical variables was occupied to compare the differences of baseline characteristics and distribution of various dietary patterns over subjects. Continuous variables were shown as means and standard deviations. Person-years (PYs) of follow-up were calculated for each subject from the date of enrolling the cohort to the date of T2D diagnosis, death, or end of follow-up. Cox proportional-hazards regression model was used to explore the associations between dietary patterns and T2D incidence. We used the Schoenfeld residuals to test the assumption of hazard proportionality in Cox regression models and found no evidence of non-proportionality. Covariates including age (18–34, 35–49, ≥50 years), BMI value (<18.5, 18.5–23.9, 24–27.9, ≥28 kg/m^2^), sex, rural (yes, no), Han Chinese (yes, no), education ( ≤ 9 years, >9 years), being married (yes, no), T2D family history (yes, no), smoking status (yes, no), alcohol use (yes, no), physical activity (yes, no), and IFG (yes, no) were adjusted in the model. In China, a BMI <18.5 means that the individuals are lean, and they may have some health problems related to eating or absorption, which have possible effects on diet habits. Also, it is likely that the long-term dietary effects have not been observed in 2 years. Therefore, sensitivity tests were conducted by excluding the individuals who were followed up <2 years or whose BMI was <18.5 at baseline. All statistical tests were two-sided (α = 0.05) and performed using R software (Version 4.0.3; R Foundation for Statistical Computing, Vienna, Austria).

## Results

### Baseline Characteristics

As shown in Table 1, more than half of the 7,023 eligible subjects were the age 18–34 years old and were women. Compared with participants free of T2D, new-onset T2D cases were older, more likely to be Han Chinese, and live in rural areas. They had a higher level of baseline BMI and a lower level of education, too. There were no significant differences in sex, marriage status, T2D family history, fasting plasma glucose levels, and lifestyle including smoking, alcohol drinking, and physical activity.

**Table 1:** Baseline characteristics among participants in Southwest China.

**Characteristics**	**Total**	**Non-T2D**	**Incident T2D**	***P*** **-value**
Participants, *n*	7,203	6,454	749	
Age, years				<0.001
18–34	3,925 (54.5)	3,594 (55.7)	331 (44.2)	
35–49	2,127 (29.5)	1,869 (29.0)	258 (34.4)	
≥50	1,151 (16.0)	991 (15.4)	160 (21.4)	
Women, %	3,829 (53.2)	3,439 (53.3)	390 (52.1)	0.554
Ethnicity (Han Chinese), %	4,209 (58.4)	3,726 (57.7)	483 (64.5)	<0.001
Education >9 years, %	3,044 (42.3)	2,779 (43.1)	265 (35.4)	<0.001
Married, %	5,783 (80.3)	5,178 (80.2)	605 (80.8)	0.759
Rural, %	4,733 (65.7)	4,206 (65.2)	527 (70.4)	0.005
Current smoker, %	2,066 (28.7)	1830 (28.4)	236 (31.5)	0.078
Alcohol use, %	2,285 (31.7)	2,033 (31.5)	252 (33.6)	0.249
Physical activity, %	6,268 (87.0)	5,607 (86.9)	661 (88.3)	0.316
T2D family history, %	91 (1.3)	80 (1.2)	11 (1.5)	0.720
IFG, %	527 (7.3)	464 (7.2)	63 (8.4)	0.255
BMI, kg/m^2^				<0.001
<18.5	437 (6.1)	401 (6.2)	36 (4.8)	
18.5–23.9	4,592 (63.8)	4,173 (64.7)	419 (56.2)	
24.0–27.9	1,716 (23.9)	1,521 (23.6)	195 (26.2)	
≥28.0	447 (6.2)	352 (5.5)	95 (12.8)	

*T2D, type 2 diabetes; IFG, impaired fasting glucose; BMI, body mass index*.

### Distribution of Dietary Patterns

In Table 2, subjects who were over 50 years old, non-Han Chinese, had shorter education years, were married, lived in rural areas, were non-smoker, non-alcohol users, without regular physical activity or T2D family history, with IFG, and lean tended to have low adherence of junk food pattern. Those who were aged from 18 to 34 years old, men, non-Han Chinese, had longer education years, lived in rural areas, smoker, alcohol user, and with IFG were more likely to have high adherence to the vegetable-grain food pattern.

**Table 2:** Tertiles of dietary patterns over subjects with different baseline characteristics in Southwest China (%).

**Characteristics**	**Junk food pattern**				**Vegetable-grain pattern**			
	**Low**	**Medium**	**High**	***P*** **-value**	**Low**	**Medium**	**High**	***P*** **-value**
Participants, *n*	2,401	2,401	2,401		2,401	2,401	2,401	
Age, years				<0.001				<0.001
18–34	1,172 (29.9)	1,267 (32.3)	1,486 (37.9)		1,189 (30.3)	1,352 (34.4)	1,384 (35.3)	
35–49	763 (35.9)	738 (34.7)	626 (29.4)		705 (33.1)	715 (33.6)	707 (33.2)	
≥50	466 (40.5)	396 (34.4)	289 (25.1)		507 (44.0)	334 (29.0)	310 (26.9)	
Sex				0.498				<0.001
Women	1,294 (33.8)	1,254 (32.8)	1,281 (33.5)		1,433 (37.4)	1,273 (33.2)	1,123 (29.3)	
Men	1,107 (32.8)	1,147 (34.0)	1,120 (33.2)		968 (28.7)	1,128 (33.4)	1,278 (37.9)	
Ethnicity (Han Chinese)				<0.001				<0.001
Yes	1,080 (25.7)	1,406 (33.4)	1,723 (40.9)		1,597 (37.9)	1,418 (33.7)	1,194 (28.4)	
No	1,321 (44.1)	995 (33.2)	678 (22.6)		804 (26.9)	983 (32.8)	1,207 (40.3)	
Education, years				<0.001				0.003
>9	710 (23.3)	944 (31.0)	1,390 (45.7)		961 (31.6)	1,007 (33.1)	1,076 (35.3)	
≤ 9	1,691 (40.7)	1,457 (35.0)	1,011 (24.3)		1,440 (34.6)	1,394 (33.5)	1,325 (31.9)	
Married				<0.001				0.155
Yes	1,932 (33.4)	1,991 (34.4)	1,860 (32.2)		1,897 (32.8)	1,941 (33.6)	1,945 (33.6)	
No	469 (33.0)	410 (28.9)	541 (38.1)		504 (35.5)	460 (32.4)	456 (32.1)	
Rural				<0.001				<0.001
Yes	1,918 (40.5)	1,545 (32.6)	1,270 (26.8)		1,342 (28.4)	1,683 (35.6)	1,708 (36.1)	
No	483 (19.6)	856 (34.7)	1,131 (45.8)		1,059 (42.9)	718 (29.1)	693 (28.1)	
Current smoker				<0.001				<0.001
Yes	569 (27.5)	739 (35.8)	758 (36.7)		617 (29.9)	689 (33.3)	760 (36.8)	
No	1,832 (35.7)	1,662 (32.4)	1,643 (32.0)		1,784 (34.7)	1,712 (33.3)	1,641 (31.9)	
Alcohol use				<0.001				<0.001
Yes	664 (29.1)	772 (33.8)	849 (37.2)		654 (28.6)	779 (34.1)	852 (37.3)	
No	1,737 (35.3)	1,629 (33.1)	1,552 (31.6)		1,747 (35.5)	1,622 (33.0)	1,549 (31.5)	
Physical activity				<0.001				0.088
Yes	1,986 (31.7)	2,100 (33.5)	2,182 (34.8)		2,062 (32.9)	2,093 (33.4)	2,113 (33.7)	
No	415 (44.4)	301 (32.2)	219 (23.4)		339 (36.3)	308 (32.9)	288 (30.8)	
T2D family history, %				<0.001				0.865
Yes	13 (14.3)	32 (35.2)	46 (50.5)		32 (35.2)	31 (34.1)	28 (30.8)	
No	2,388 (33.6)	2,369 (33.3)	2,355 (33.1)		2,369 (33.3)	2,370 (33.3)	2,373 (33.4)	
IFG				0.016				<0.001
Yes	203 (38.5)	172 (32.6)	152 (28.8)		143 (27.1)	168 (31.9)	216 (41.0)	
No	2,185 (32.9)	2,218 (33.4)	2,241 (33.7)		2,246 (33.8)	2,220 (33.4)	2,178 (32.8)	
BMI, kg/m^2^				<0.001				0.712
<18.5	157 (35.9)	141 (32.3)	139 (31.8)		157 (35.9)	145 (33.2)	135 (30.9)	
18.5–23.9	1,612 (35.1)	1,505 (32.8)	1,475 (32.1)		1,521 (33.1)	1,519 (33.1)	1,552 (33.8)	
24.0–27.9	506 (29.5)	597 (34.8)	613 (35.7)		560 (32.6)	588 (34.3)	568 (33.1)	
≥28.0	122 (27.3)	152 (34.0)	173 (38.7)		158 (35.3)	147 (32.9)	142 (31.8)	

*T2D, type 2 diabetes; IFG, impaired fasting glucose; BMI, body mass index*.

### Dietary Patterns and Type 2 Diabetes Incidence

During 50,763.64 person-years of follow-up, 749 incident T2D cases were documented and the incidence was 14.75/1,000 person-years. As shown in Table 3, the junk food pattern was associated with type 2 diabetes incidence. Compared with individuals in the medium score category of junk food pattern, those with the lowest scores got a lower risk of incident T2D after adjusting for potential confounders (HR: 0.72, 95% CI: 0.60, 0.87). Adjustment for lifestyles including smoking, alcohol drinking, and sport attenuated the association a little, but it still remained significant (HR: 0.72, 95% CI: 0.61, 0.87). An inverse association was observed between vegetable-grain pattern and incident T2D after multivariable adjustment. Participants at the high score category of the vegetable-grain pattern had a 20% lower risk of T2D (HR: 0.80, 95% CI: 0.67, 0.95) than those at the medium intake category and this protective effect persisted after further adjusting lifestyles (HR: 0.80, 95% CI: 0.67, 0.95).

**Table 3:** The incidence risk of type 2 diabetes (T2D) over tertiles of dietary patterns among the adult population in Southwest China.

	**Cases, *n***	**Incident density/1000 PYs**	**HR (95%CI)**		
			**Model 1**	**Model 2**	**Model 3**
**Junk food pattern**					
Low	216	12.63	0.71 (0.59, 0.85)**	0.72 (0.60, 0.87)**	0.72 (0.61, 0.87)**
Medium	282	16.75	1.00	1.00	1.00
High	251	14.92	0.91 (0.77, 1.08)	0.94 (0.79, 1.12)	0.93 (0.78, 1.11)
**Vegetable and grain pattern**					
Low	234	13.93	0.84 (0.71, 1.01)	0.85 (0.71, 1.01)	0.86 (0.72, 1.02)
Medium	289	16.95	1.00	1.00	1.00
High	226	13.37	0.79 (0.66, 0.94)**	0.80 (0.67, 0.95)*	0.80 (0.67, 0.95)*

*Model 1: adjusted for age, sex*.

*Model 2: model 1 plus location, ethnicity, education, marriage, T2D family history, and baseline BMI value*.

*Model 3: model 2 plus smoking status, alcohol use, regular physical exercise, and IFG*.

** *P < 0.001*,

* *P < 0.05*.

*PY, person-years; HR, hazard ratio; 95% CI, 95% confidence interval*.

**Figure 1:**
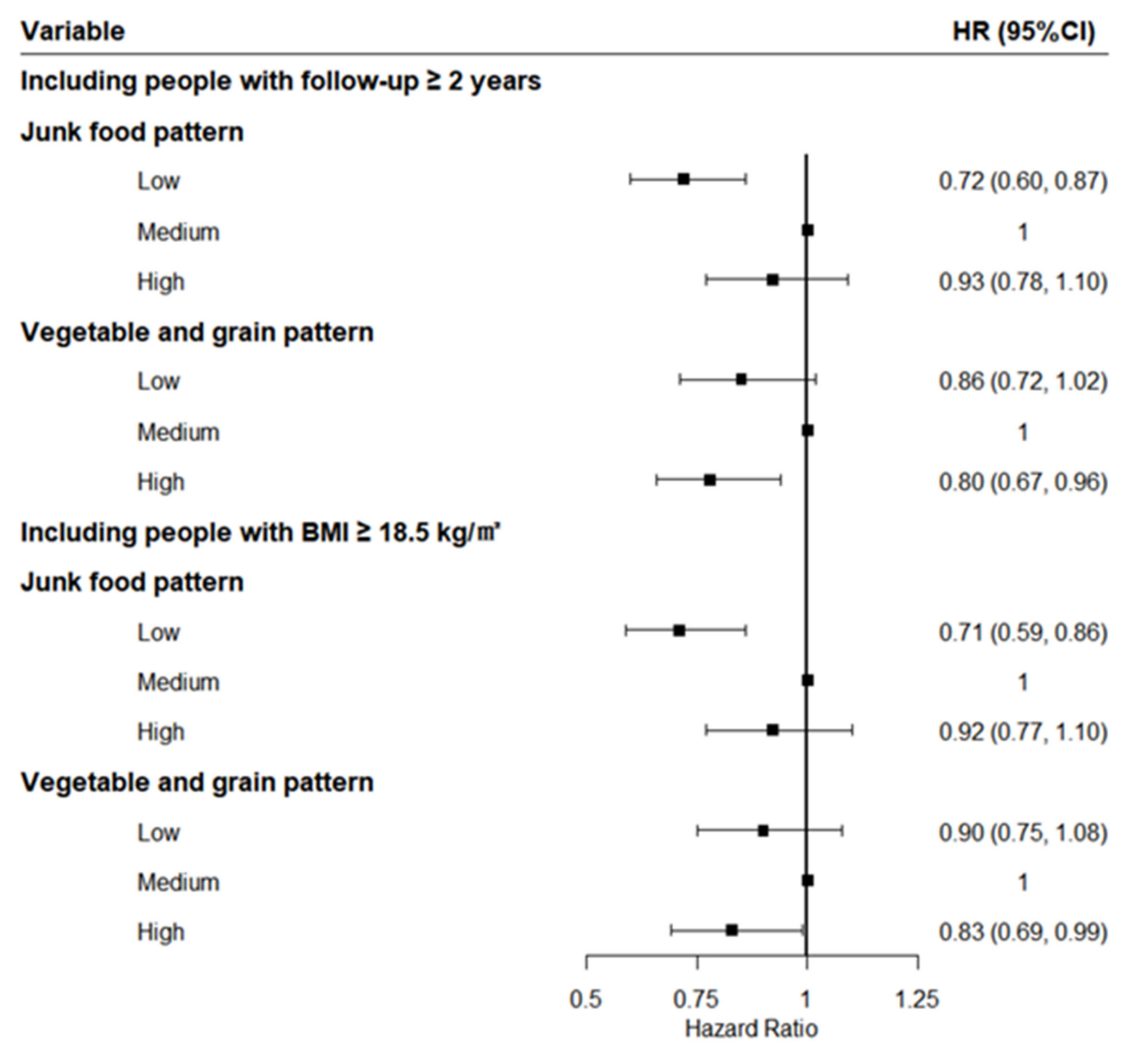
Sensitivity analysis after excluding those who entered the cohort within 2 years or were lean.

Those findings remained robust in the sensitivity analysis. The protective effects of the highest score of vegetable-grain pattern and the lowest score of junk food pattern were virtually unchanged after excluding those who entered the cohort within 2 years or belonged to the lean one (BMI <18.5) at baseline (Figure 1).

### Associations Between Dietary Patterns, and Averages of Follow-Up Fasting Plasma Glucose and Their Changes

Further, potential associations between different dietary patterns, and averages of FPG at follow-up and their changes between baseline and follow-up were explored among non-diabetic participants. For the junk food pattern, the follow-up FPG and its rising change increased with the incremental score of this pattern statistically, and significant inverse trends were observed over vegetable-grain pattern scores (Table 4).

**Table 4:** Associations between different dietary patterns, and averages of follow-up fasting plasma glucose and its changes among non-diabetic participants (mmol, Mean ± SD).

	**Low**	**Medium**	**High**	***P*-trend**
**Follow-up FPG**
Junk food pattern	5.18 ± 0.68	5.24 ± 0.70	5.28 ± 0.69	<0.001
Vegetable-grain pattern	5.28 ± 0.70	5.25 ± 0.70	5.17 ± 0.67	<0.001
**FPG change**
Junk food pattern	0.05 ± 1.01	0.23 ± 1.02	0.30 ± 1.00	<0.001
Vegetable-grain pattern	0.29 ± 1.01	0.21 ± 1.02	0.08 ± 1.01	<0.001

*FPG, fasting plasma glucose*.

## Discussion

Studies regarding the association of empirically derived dietary patterns and the risk of T2D in Southwest China were sparse so far. This prospective study identified two major dietary patterns by factor analysis, and both the junk food pattern and vegetable-grain pattern were significantly associated with T2D among the study population in Southwest China. Those findings remained robust in the sensitivity analysis among those followed up for 2 years or longer and with a BMI of 18.5 or greater at baseline. Moreover, significant positive linear trends were observed for associations between junk food pattern, and averages of follow-up FPG and its changes, and inverse linear trends existed in the associations between vegetable-grain pattern, and averages of follow-up FPG and its changes. Those results were in agreement with previous studies reporting that various dietary patterns were related to incident T2D (18, 19). They suggested that a healthy diet pattern may benefit the prevention and control of T2D and some interventions should be developed to improve the diet behaviors.

In some researches, fruit juices, sweetened beverages, sweets, and desserts had been proved to be less healthy plant foods for diabetes (19, 20). A meta-analysis based on 5 studies suggested that “unhealthy” dietary pattern like consuming higher amounts of high-fat dairy and fried products was associated with a 44% increased risk of diabetes (4). In this study, low intake of junk food pattern, characterized by high consumption of fried food, soft drinks, and desserts resulted in a 28% reduction in risk of T2D compared with medium intake. Dietary components in such a pattern that may affect T2D risk included higher saturated fat and glycemic load from high intakes of snacks and desserts (21, 22), as part of the western pattern's unhealthy constituents. The high glycemic load had been documented to adversely affect insulin sensitivity, a well-established detrimental factor for diabetes (23, 24). On the one hand, a high level of added sugar and calorie content strongly increases the risk for diabetes with weight gain, worse metabolism of glucose, and lower insulin sensitivity (25, 26); on the other hand, advanced glycation of high-fat products can contribute to enhanced oxidative stress and higher inflammatory markers that may be accompanied with insulin resistance (27). But the increased risk was not observed significantly in the high level of such a pattern. One of the possible reasons was that the intake did not reach the dangerous thresholds in the study population. Another was that the sample size was not enough for such association in this study.

Also, the vegetable-grain pattern was observed a protective effect on incident T2D in this study, with a 20% lower T2D risk, which was similar to the results from a previous systematic review and meta-analysis (28). Jordi et al. provided evidence on the inverse relationship between adherence to a plant-based dietary pattern and the risk for developing T2D, too (5). Several potential mechanisms may explain the observed favorable association. Firstly, whole grains and vegetables may bring a rich array of dietary fiber, which has been proved in several prospective studies to reduce levels of inflammatory markers (29, 30). Secondly, one previous study in Chinese patients with diabetes has recognized increasing fiber and rich antioxidant intake from vegetables as an effective approach to lower HbA1c levels (31) and further reduce T2D risk. Thirdly, whole grains, tubers, and vegetables in this pattern had a low glycemic index and may help to improve insulin sensitivity, and thereby enhance glycemic control (19). Of course, it is most likely a combination of dietary factors responsible for the robust associations. However, the findings remain controversial. Contrary to our results, Erber et al. showed that the vegetable pattern did not confer a lower risk for diabetes in women (32). Another previous research also reported refined grains and meat patterns to be a major risk factor for diabetes (10). Although the vegetable-grain pattern in this study shared some components with other patterns, no meat was contained and the concrete effect partly depended on the proportion and category of food items, like the whole or refined grains (23), and dark or light vegetables. The complex difference in components of different patterns may be due to the diversity in race, age, culture, and lifestyle over the different study populations.

To the best of our knowledge, there were few studies on dietary patterns derived from factor analysis in Southwest China. This study highlights the various effects on T2D of different dietary patterns which may be targeted for local people and emphasizes the importance of considering the quality of foods consumed for diabetes. This study had other strengths. On the one hand, this prospective cohort study filled the gap of the association between dietary patterns and T2D risk in Southwest China among diverse minority groups with different food preferences, and those findings offered further insights into the dietary structure for T2D prevention among the adult Chinese population. On the other hand, dietary pattern beyond a single nutrient or food is easier to make recommendations for the local population and improve dietary behaviors for local residents. The limitations of this study should also be noted. First, the baseline covariates information used in the analysis might lead to residual confounding if those covariates may be time-varying. Second, dietary pattern analysis requires subjective decisions in naming the food pattern although some previous studies can be referred to. Third, the effects of different foods may be related to cooking ways, which were not considered in this study. In addition, diet information was self-reported by subjects, and measurement errors were inevitable which may bias the findings from this study.

## Conclusion

Higher adherence to the vegetable-grain pattern and lower adherence to the junk food pattern significantly lowered T2D incidence among the Chinese population in Southwest China. Those findings added evidence on the recommendation of healthy dietary patterns to prevent incident T2D for the Chinese adult population in China, especially in Southwest China. Some interventions and policies should be developed and implemented to improve the diet behaviors to prevent and control T2D better among the Chinese population in the future.

## Data Availability Statement

The original contributions presented in the study are included in the article/Supplementary Material, further inquiries can be directed to the corresponding authors.

## Ethics Statement

The studies involving human participants were reviewed and approved by the Institutional Review Board of Guizhou Province Center for Disease Control and Prevention (No. S2017-02). The patients/participants provided their written informed consent to participate in this study.

## Author Contributions

LX and YW conceptualized, designed and conducted the study with the help of NW, LZ, FZ, KX, TL, and CF. LX and YW wrote the first draft of the manuscript. NW, LZ, FZ, KX, TL, and CF contributed significantly to the revision of the manuscript. All authors read and approved the final manuscript, had full access to all the data in the study, and accept responsibility for the decision to submit for publication.

## Funding

This work was supported by the Guizhou Province Science and Technology Support Program (Qiankehe [2018]2819).

## Conflict of Interest

The authors declare that the research was conducted in the absence of any commercial or financial relationships that could be construed as a potential conflict of interest.

## Publisher's Note

All claims expressed in this article are solely those of the authors and do not necessarily represent those of their affiliated organizations, or those of the publisher, the editors and the reviewers. Any product that may be evaluated in this article, or claim that may be made by its manufacturer, is not guaranteed or endorsed by the publisher.
