# Supplementary material for: Associations of Dietary Patterns and Incident Type 2 Diabetes in a Community Population Cohort From Southwest China
Source: Front Public Health. 2022 Feb 3;10:773172. doi: 10.3389/fpubh.2022.773172 (PMC8850375; doi:10.3389/fpubh.2022.773172)
Supplement: Supplementary file 1 [file Data_Sheet_1.docx]

Supplementary Material

**Table S1. Factor loading matrix after dietary pattern rotation and average daily intake of each food group (g)**

|  | **Junk food pattern** | | | | **Vegetable-grain pattern** | | | |
| --- | --- | --- | --- | --- | --- | --- | --- | --- |
| **Food category** | Low | Medium | High | Factor loading | Low | Medium | High | Factor loading |
| Grain (rice, flour and coarse cereals, etc) | 448.20 | 414.42 | 399.87 | -0.03 | 420.83 | 401.19 | 574.99 | **0.42** |
| Potato (potato, taro and sweet potato, etc) | 7.77 | 21.04 | 38.43 | 0.21 | 21.28 | 20.25 | 25.71 | 0.05 |
| Meat (pork, beef, mutton, chicken, duck, etc) | 66.49 | 101.97 | 135.54 | 0.14 | 64.52 | 85.84 | 153.64 | 0.27 |
| Fish (fish, shrimp, crab, etc) | 2.46 | 5.49 | 16.73 | 0.12 | 4.86 | 7.09 | 12.72 | 0.09 |
| Vegetable (light- and dark-colored vegetables) | 460.70 | 340.93 | 365.51 | -0.04 | 195.26 | 352.29 | 619.60 | **0.54** |
| Fruit (apple, banana, etc) | 26.59 | 51.37 | 92.57 | 0.15 | 35.38 | 52.14 | 83.00 | 0.20 |
| Egg | 8.91 | 14.20 | 22.36 | 0.11 | 18.55 | 13.66 | 13.26 | -0.08 |
| Milk | 1.01 | 4.63 | 31.15 | 0.17 | 16.51 | 10.34 | 9.94 | -0.04 |
| Legume (soybean, mung, etc) | 24.40 | 20.94 | 33.41 | 0.07 | 22.81 | 24.65 | 31.28 | 0.07 |
| Fried food (seedcake, deep-fried dough stick, etc) | 0.17 | 1.22 | 10.98 | **0.34** | 3.79 | 4.45 | 4.12 | 0.00 |
| Soft drinks (fruit beverage, carbonated drinks, etc) | 0.63 | 4.66 | 70.98 | **0.54** | 26.05 | 26.25 | 23.97 | -0.02 |
| Dessert (bread, cake, yolk pie, etc) | 0.21 | 1.23 | 13.76 | **0.47** | 7.08 | 4.73 | 3.40 | -0.06 |
| Oil (soybean oil, olive oil, etc) | 33.98 | 46.94 | 52.64 | 0.08 | 44.54 | 46.43 | 42.59 | -0.03 |
| Pickles | 7.39 | 4.97 | 6.70 | -0.01 | 5.34 | 5.57 | 8.15 | 0.06 |
| Sufu | 1.72 | 0.95 | 1.76 | 0.01 | 1.99 | 1.14 | 1.31 | -0.04 |
| Soybean paste | 0.99 | 0.97 | 1.72 | 0.04 | 1.65 | 0.93 | 1.10 | -0.04 |


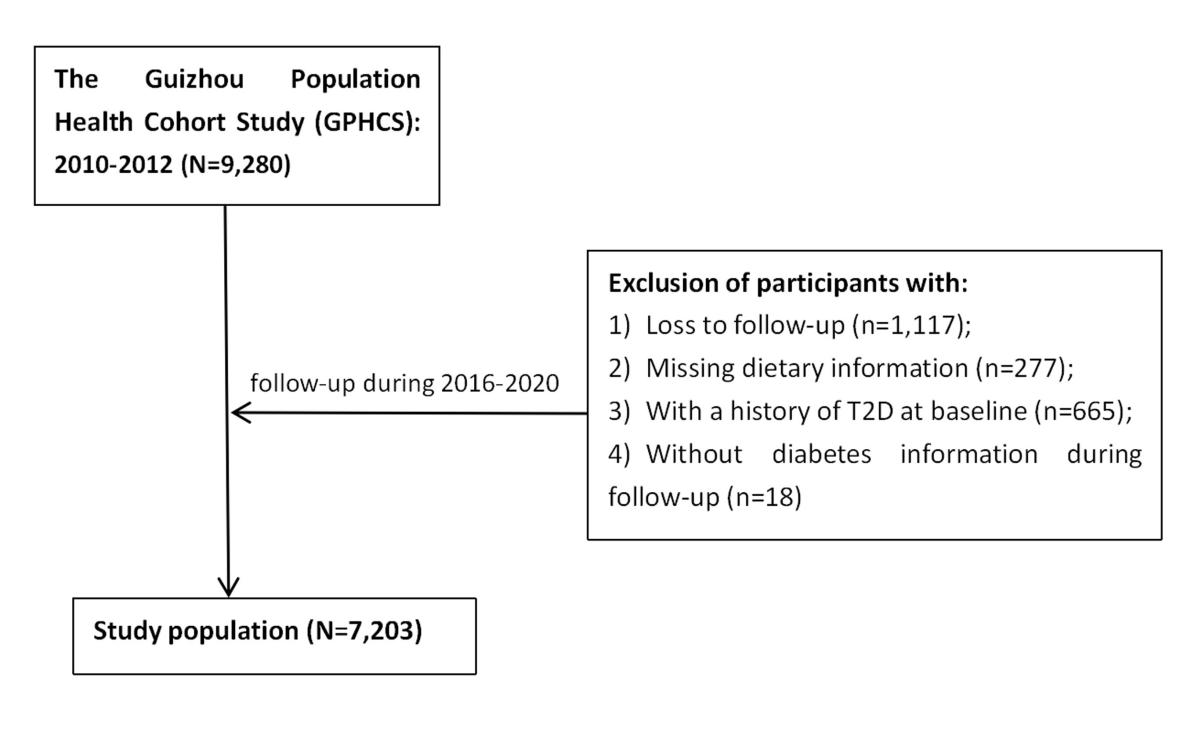


**Supplementary Figure 1. The flow chart of the cohort study**
